# Fibrosis-4 Model Influences Results of Patients with Hepatocellular Carcinoma Undergoing Hepatectomy

**DOI:** 10.1155/2018/4305408

**Published:** 2018-07-02

**Authors:** Haiqing Wang, Aixiang Liu, Wentao Bo, Xielin Feng, Yong Hu, Lang Tian, Hui Zhang, Xiaoli Tang, Lixia Zhang

**Affiliations:** Department of Hepato-Biliary-Pancreatic Surgery, Sichuan Cancer Hospital & Institute, Sichuan Cancer Center, School of Medicine, University of Electronic Science and Technology of China, Chengdu, China

## Abstract

**Background:**

Several noninvasive models based on routine laboratory index have been developed to predict liver fibrosis. Our aim is to discuss whether these indexes could predict prognosis in patients with hepatocellular carcinoma undergoing hepatectomy.

**Methods:**

This study retrospectively enrolled 788 consecutive hepatocellular carcinoma patients undergoing liver resection in the cohort. Univariate and multivariate analysis were used to identify the risk factors of complications, survival, and disease-free survival.

**Results:**

Fibrosis-4 index had the best prediction ability for cirrhosis among other noninvasive models. Both the univariate and multivariate analyses showed that fibrosis-4 was independent risk factor for survival and disease-free survival. With the optimal cutoff value of 3.15, patients with fibrosis-4 *⩾*3.15 had higher postoperative hepatic insufficiency (P=0.006) and worse survival than the fibrosis-4<3.15 group. The corresponding 1-year, 3-year, and 5-year overall survival were 80.9%, 56.3%, and 44.6% in the High fibrosis-4 group and were 86.5%, 69.9%, and 63.2% in the Low fibrosis-4 group, respectively (P<0.001). Worse disease-free survival was also observed in the fibrosis-4 *⩾*3.15 group; the corresponding 1-year, 3-year, and 5-year disease-free survival were 74.9%, 45.3%, and 24.6% for the fibrosis-4 *⩾*3.15 group and were 81.8%, 54.9%, and 34.4% for the fibrosis-4<3.15 group (P=0.009).

**Conclusions:**

Fibrosis-4 is useful for assessing the short-term and long-term results for hepatocellular carcinoma patients with liver resection.

## 1. Introduction

Hepatocellular carcinoma (HCC) represents the fifth most common tumor and the third leading cause of global cancer mortality [1, 2]. Radical liver resection and liver transplantation currently represent the most effective treatment procedures for HCC [3]. Refinement of surgical interventions, perioperative management, and earlier diagnosis in liver surgery over recent decades have resulted in marked improvement of long-term survival for HCC patients undergoing hepatectomy. However, the 25% annual recurrence rate is high, with the 5-year recurrence rate exceeding 60%[2]. Liver fibrosis and cirrhosis are not only critical risk factors in the development of HCC, but also the most significant risk factors for HCC recurrence and postoperative complications. In addition, almost 80% of HCCs are correlated with hepatitis B (HBV) or hepatitis C (HCV) viral infections and varying degrees of liver fibrosis or cirrhosis [4]. Therefore, it is essential to identify liver fibrosis risk factors in order to optimize rational treatments. However, identification of liver fibrosis and cirrhosis requires physically intrusive biopsy or pathology examinations, which are also associated with significant costs and risks of bleeding. Recently, a number of studies have reported the existence of noninvasive biomarkers, detectable within routine laboratory testing, which are able to accurately forecast the presence of liver fibrosis and also have the capability to differentiate between good and poor prognoses for patients. Examples of such noninvasive biomarkers include aspartate aminotransferase-to-alanine transaminase (AST/ALT), aspartate aminotransferase-to-platelet ratio index (APRI), fibrosis-4 index, Forn's index, and Lok index [5–11]. However, the conclusions from these studies remain controversial. Whilst some studies reported that fibrosis-4 [8, 10, 12–14] provided optimum discrimination capability with respect to liver cirrhosis and also appeared to offer capabilities with respect to predicting long-term outcomes, the study conducted by Choi [6] indicated that the Forn index was the optimum biomarker, whilst Pang [11] identified King's score as the optimum option. Therefore, the aim of this paper is to determine whether such noninvasive models have the ability to predict cirrhosis, postoperative complications, and survival in patients presenting with HCC and undergoing hepatectomy with R0 intent and also to determine the optimum approach.

## 2. Materials and Methods

### 2.1. Study Population

Between January 2009 and January 2016, the 788 consecutive HCC patients undergoing R0 liver resection in our institute were included in this study. HCC patients with obstructive jaundice, palliative resection, and metastatic hepatic carcinoma were excluded from the study. The study protocol obtained prior approval from the Clinical Research Ethics Committee of the hospital (research registry 3069) and the research has been reported in accordance with STROCSS guidelines [15]. Written informed consent was obtained from all patients according to the policies of the committee. Medical records detailing patient demographics, comorbid conditions, laboratory values, intraoperative parameters, and postoperative outcomes were sourced from a prospectively maintained database. For all patients, liver resection was performed by the same surgical team and every patient included in the study had had their diagnosis for HCC confirmed via postoperative histology testing.

### 2.2. Perioperative Management and Follow-Up

All patients were managed by the identical surgical team. Patients gave a detailed case history and underwent thorough physical and routine preoperative laboratory examination. Routine preoperative imaging examination to assess tumor extent included contrast computed tomography or magnetic resonance imaging of the abdomen. Echocardiography, chest radiography (or computed tomography), and pulmonary function tests were performed if required. Patients underwent general anesthesia prior to operation and were explored via an extended right subcostal incision, with routine intraoperative ultrasonography being employed. Hemihepatic vascular inflow occlusion or the Pringle manoeuver was employed according to the surgeon's preference in most patients. Liver parenchymal transection was performed using harmonic scalpel or Cavitron ultrasonic surgical aspirator. Follow-up consisted of ultrasonography, alpha-fetoprotein (AFP), and computed tomography or magnetic resonance imaging performed every 3 months at our institution to monitor for potential HCC recurrence and metastasis. If recurrence was confirmed, patients underwent additional treatment for recurrent HCC according to standard treatment guidelines [3].

### 2.3. Definition of the Parameters and Calculation of the Noninvasive Index

The Clavien-Dindo complication classification system was used for postoperative complication grading [16]. Posthepatectomy liver insufficiency was defined according to the criterion of International Study Group of Liver Surgery [17]. Assessments for liver tumor and fibrosis assessment were conducted by two pathologists in our hospital and stages of fibrosis stages for the nonneoplastic liver tissue were evaluated via the Ishak score system, with cirrhosis being defined as an Ishak fibrosis stage of F5-F6 [18]. The degree of differentiation of HCC in each resected specimen was determined in addition to differentiated, moderately differentiated, and poorly differentiated HCC. All noninvasive indices were calculated based upon laboratory data one week prior to liver resection. APRI was calculated as (AST/UNL)/platelet (10^9^/L) × 100. Fibrosis-4 was calculated as age × AST/[platelet × (ALT)^1/2^]. Lok index was calculated as 5.56 – 0.0089 × (platelet count) + 1.26 × (AST/ALT) + 5.27 ×international normalized ratio (INR). King Score was calculated as age× AST×INR/platelet [6, 11, 12, 19].

### 2.4. Statistical Analysis

All statistical analyses were performed using SPSS Version 17 statistical software, and statistical significance was set at* P* < 0.05. Continuous variables were reported as mean (SD) or median (range) and were compared either using the Student* t*-test for continuous variables with parametric distribution or using Mann–Whitney U or Kruskal–Wallis H tests for those exhibiting nonparametric distributions. Categorical variables were reported as numbers and percentages and compared using Pearson's *χ*2 analysis or Fisher's exact test. The predictive ability of noninvasive indices for cirrhosis was assessed by the receiver employing a characteristic (ROC) curve and calculating the relevant area under the ROC (AUROC) curve. The optimal cutoff value was set as the value maximizing the sum of sensitivity and specificity, i.e., the Youden index [20]. The cumulative overall survival and disease-free survival rates were calculated using the Kaplan–Meier method, tested via the log-rank test. The Cox proportional hazards model was utilised for univariate and multivariate analysis of factors related to survival and disease-free survival.

## 3. Results

### 3.1. Patient Characteristics and Noninvasive Index

**Figure 1:**
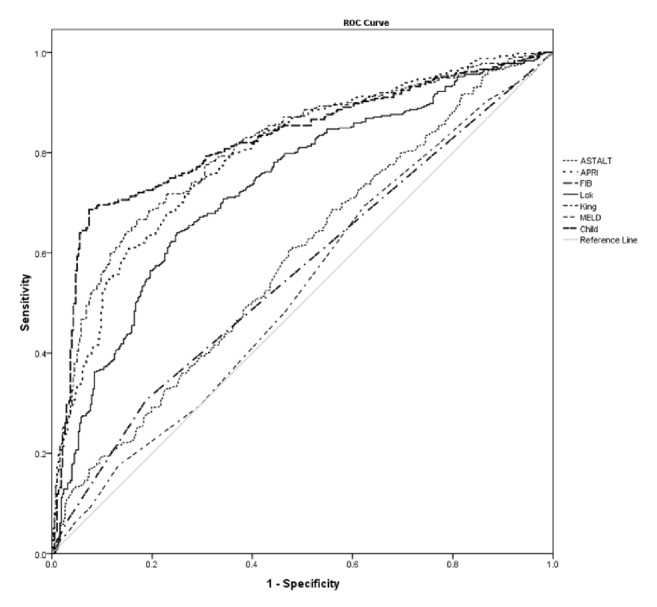
Receiver operating characteristic curve for King score, APRI, Lok index, AST/ALT, Child-Pugh score, and fibrosis-4 in relation to cirrhosis.

A total of 788 consecutive HCC patients were included in this study and the average age was 51.1±11.9. 84.4% of patients were HBV-related HCC, and 94.7% had Child-Pugh score A. According to histologic pathology, 52.2% of the patients were diagnosed with cirrhosis. 19.3% of the patients had multiple types of tumors at diagnosis, and macrovascular invasion was observed by image examination in 11.8% of the patients (Table 1). According to univariate analyse, HBV, HBV-DNA, age, Child-Pugh score, AST, ALT, PLT, fibrosis-4, AST/ALT, APRI, Lok index, King score, and albumin were associated with cirrhosis. However, only HBV, HBV-DNA, Child-Pugh score, fibrosis-4, and AST/ALT were the independent risk factors for cirrhosis by multivariate analyses. Based on AUROC curve, fibrosis-4 showed the best ability to predict cirrhosis (AUROC=0.824, P<0.001) among King score (AUROC=0.807, P<0.001), APRI (AUROC=0.795, P<0.001), Lok index (AUROC=0.731, P<0.001), AST/ALT (AUROC=0.586, P<0.001), MELD score (AUROC=0.528, P=0.171), and Child-Pugh score (AUROC=0.561, P=0.003) (Figure 1). Thus, fibrosis-4 was an ideal and independent index for cirrhosis prediction. The optimal cutoff for fibrosis-4 to predict cirrhosis was 3.15, maximizing the Youden index to 0.612 (sensitivity= 0.686, specificity = 0.93). The median follow-up period was 28 months. The overall survival rates of included patients at 1-year, 3-year, and 5-year were 84.2%, 64.0%, and 55.6%, respectively. Meanwhile, the overall disease-free survival rates of included patients at 1-year, 3-year, and 5-year were 79.0%, 50.7%, and 31%, respectively.

### 3.2. Univariate and Multivariate Analyses of Prognostic Factors for Overall Survival

The results of the Cox regression hazards model for predictors of overall survival were shown in Table 2. Univariate analysis identified that cirrhosis (OR=1.3,95%CI:1.01-1.71), Barcelona Clinic Liver Cancer algorithm (BCLC) B-C stage (P<0.02), multiple tumor (OR=3.55,95%CI:1.12-2.1), macrovascular invasion (OR=3.55,95%CI:2.6-4.8), microvascular invasion (OR=1.9,95%CI:1.4-2.5), complications III-V (OR=1.7,95%CI: 1.14-2.54), and all the five noninvasive indexes—AST/ALT (OR=1.15,95%CI:1.07-1.23), APRI (OR=1.08,95%CI:1.02-1.13), fibrosis-4 (OR=1.04,95%CI:1.02-1.07), Lok index (OR=1.11,95%CI:1.05-1.17), and King score (OR=1.01,95%CI:1.01-1.01)—were the risk factors for overall survival. According to the multivariate analysis, only BCLC B-C stage (P<0.03), microvascular invasion (OR=1.5,95%CI: 1.11-2.01), complications III-V (OR=1.6,95%CI: 1.08-2.44), and fibrosis-4(OR=1.03,95%CI: 1.03-1.08) were independent risk factors for overall survival (Table 2). The noninvasive indexes such as AST/ALT, APRI, Lok index, and King score were not independent factors of overall survival for HCC patients with R0 liver resection.

### 3.3. Univariate and Multivariate Analyses of Prognostic Factors for Overall Disease-Free Survival

Univariate analysis (Table 3) showed that 12 variables, including fibrosis-4, were significantly associated with the overall disease-free survival. The other 11 variables were cirrhosis, BCLC C stage, multiple tumor, macrovascular invasion, microvascular invasion, AFP, tumor differentiation, AST/ALT, APRI, Lok index, and King score. These significantly different variables were included in a Cox regression model to identify whether fibrosis-4 was an independent risk factor for disease-free survival. The Cox regression analysis indicated that fibrosis-4, BCLC stage, and tumor differentiation were independent risk factors for disease-free survival.

### 3.4. The Comparison of Fibrosis-4 <3.15 Group and Fibrosis-4*⩾*3.15 Group

**Figure 2:**
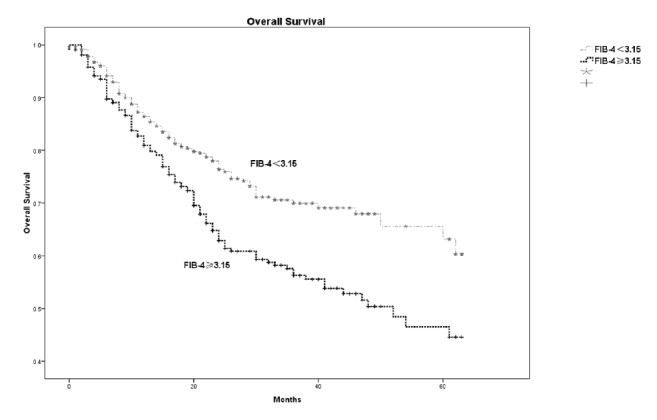
The 1-year, 3-year, and 5-year overall survival between fibrosis-4 ≥3.15 group and fibrosis-4<3.15 group. Fibrosis-4 ≥3.15 group had worse survival than the fibrosis-4<3.15 group, with the corresponding 1-year, 3-year, and 5-year overall survival of 80.9%, 56.3%, and 44.6% for fibrosis-4 ≥3.15 group and 86.5%, 69.9%, and 63.2% for fibrosis-4<3.15 group (P<0.001).

**Figure 3:**
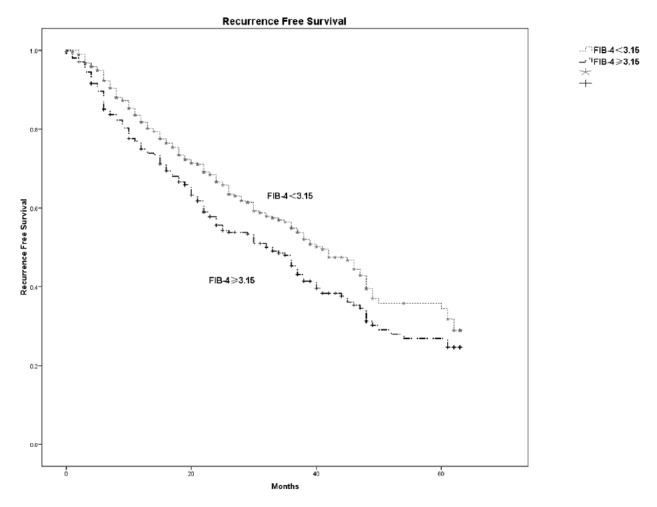
The 1-year, 3-year, and 5-year disease-free survival between fibrosis-4 ≥3.15 group and fibrosis-4<3.15 group. Worse disease-free survival was also observed in the fibrosis-4 *⩾*3.15 group; the corresponding 1-year, 3-year, and 5-year disease-free survival were 74.9%, 45.3%, and 24.6% for fibrosis-4 *⩾*3.15 group and 81.8%, 54.9%, and 34.4% for the fibrosis-4<3.15 group (P=0.009).

With the cutoff value of 3.15, patients were stratified into the Low fibrosis-4 group (477 patients) and High fibrosis-4 group (311 patients).  Table 4 compared the characteristics of the two groups. The High fibrosis-4 group had higher mean age (56.3±10.4 versus 47.7±11.7, P<0.01), higher MELD score (P=0.001), and more patients with Child-Pugh grade B (7.4% versus 4.0%, P=0.037). In addition, the High fibrosis-4 group had worse liver function, including higher AST, ALT, and INR, and lower albumin and platelet (PLT). More postoperative hepatic insufficiency (11.9% versus 6.3%, P=0.006) was observed in the High fibrosis-4 group and longer hospital stay existed in the High fibrosis-4 group (Table 4). The High fibrosis-4 group also had worse overall survival and disease-free survival. The corresponding 1-year, 3-year, and 5-year overall survival were 80.9%, 56.3%, and 44.6% in the High fibrosis-4 group and were 86.5%, 69.9%, and 63.2% in the Low fibrosis-4 group, respectively (P<0.001) (Figure 2). The corresponding 1-year, 3-year, and 5-year disease-free survival were 74.9%, 45.3%, and 24.6% for the High fibrosis-4 group and 81.8%, 54.9%, and 34.4% for the Low fibrosis-4 group (P=0.009) (Figure 3).

## 4. Discussion

In this study, it was identified that noninvasive indices, such as the King score, APRI, Lok index, AST/ALT, and fibrosis-4, possess good prediction ability for cirrhosis; however, the fibrosis-4 index offers the optimum predictive abilities for cirrhosis, with an AUROC of 0.842. The univariate and multivariate analyses demonstrated that the fibrosis-4 index, rather than other noninvasive indices, provided the best independent prognostic factor for overall survival and disease-free survival for HCC patients with R0 liver resection. For an optimal cutoff value of 3.15, patients exhibiting a fibrosis-4 index greater than 3.15 exhibited poorer liver function and consequently greater postoperative hepatic insufficiency. In addition, a fibrosis-4 index greater than 3.15 also indicated poorer rates of overall survival and disease-free survival.

In each case, liver function was assessed by the Child-Pugh score prior to liver resection; however, this index was not sufficiently sensitive to highlight liver damage [3]. However, underlying liver injury, such as liver fibrosis and cirrhosis, could still appear with a positive Child-Pugh score. The present study indicated that 94.7% of patients gave a Child-Pugh score of A; however, 52.2% of these also exhibited cirrhosis from pathological histology results. Moreover, fibrosis and cirrhosis were demonstrated to be independent risk factors for HCC carcinogenesis and recurrence. Therefore, the Child-Pugh score cannot be considered appropriate for accurate evaluation of liver function and survival, with a more precise index being required. Therefore, many studies have attempted to screen noninvasive markers in order to reflect liver fibrosis, e.g., the King score, APRI, Lok index, AST/ALT, and fibrosis-4. These models were initially proposed as valid methodologies for the prediction of liver fibrosis in patients with chronic hepatitis B and C [21, 22] and then subsequently utilised to predict tumor recurrence in patients with HCC [6, 11–13, 23]. However, evidence for which index represents the best predictive ability is inconclusive. The present study indicated that fibrosis-4 not only demonstrated the best predictive ability for cirrhosis, but also was strongly associated with rates for future survival and recurrence. These results for the Fibrosis-4 marker are self-explanatory. Fibrosis-4 comprises four discrete parameters: ALT, AST, age, and PLT. All the four parameters are directly associated with a poor prognosis. Firstly, a number of studies have proposed that elderly HCC patients have a greater potential to present with cirrhosis than younger patients [24, 25]. This may be due to elderly patients having a longer history of HBV or HCV infection and subsequently a greater opportunity to develop cirrhosis. Secondly, elevated ALT and AST levels reflect the existing liver damage and active liver inflammation. Chronic necroinflammation and hepatocellular regeneration in cirrhosis liver lead to the production of oxygen free radicals, chromosomal mutations, and malignant transformation of proliferating hepatocytes and eventually contribute to tumor recurrence in the remnant liver [6, 26]. Thirdly, thrombocytopenia is common in HCC patients and may be explained by portal hypertension leading to pooling of platelets in an enlarged spleen, typically associated with cirrhosis [3]. Many studies [27, 28] have identified platelet involvement in inflammatory responses, liver regeneration, and the regulation of angiogenesis, with additional studies demonstrating that platelet count and platelet-based models represent effective tools for the prediction of postoperative HCC recurrence, particularly late recurrence. Consequently, the fibrosis-4 model offers the potential ability to influence prognosis of HCC patients and represents a potential independent prognostic factor for both overall survival and disease-free survival.

In addition to fibrosis-4, other predictive prognosis risk factors include the HCC BCLC staging system, microvascular invasive complications, and also postoperative complications, which contribute a more significant risk factor for HCC prognosis than fibrosis-4. Consequently, it can be seen that the most significant risk factors for HCC recurrence and survival in the present study were represented by tumor-related factors, with such factors also been demonstrated to be positively associated with HCC recurrence [6, 12, 13]. However, it was considered anomalous that fibrosis-4, rather than cirrhosis, represented the independent prognostic factor for both survival and disease-free survival of HCC patients. This is thought to be due to the fact that liver fibrosis and necroinflammation exhibit greater severity at HCC peripheries than within the liver parenchyma bordering the tumor. The nonneoplastic liver within the surgical resection specimen cannot represent that of the remnant liver. Hence, the fibrosis-4 index, rather than cirrhosis, is potentially more able to accurately represent the underlying liver damage [6, 29].

As identified in previous studies [8, 10, 12–14, 19] on noninvasive markers for prediction prognosis, the present study also concludes that fibrosis-4 is able to offer a better overall prediction with regard to survival and disease-free survival for HCC patients subjected to liver resection. In addition, a further difference was observed in the present study. Studies by Dong [7, 23] identified that fibrosis-4 was associated with both complications and hepatic insufficiency. However, the present study revealed that fibrosis-4 only influenced postoperative hepatic insufficiency, not general complications. Based on an optimal cutoff value of 3.15, patients were segregated into a group with a fibrosis-4 index greater than 3.15 and a group with a fibrosis-4 index less than 3.15. The group with fibrosis-4 greater than 3.15 exhibited poorer liver function (with a Child-Pugh score of B) presenting with high INR, AST, and ALT levels and lower albumin and PLT levels. This is considered to be predominant due to the fact that patients exhibiting higher fibrosis-4 also exhibited severe cirrhosis. This is considered to be the primary reason that patients with a fibrosis-4 index greater than 3.15 exhibited greater hepatic insufficiency and required a longer hospital stay in the present study. A secondary reason included the fact that patients with a fibrosis-4 index greater than 3.15 also exhibited a lower PLT level. Collated data from experimental and clinical studies has demonstrated the significant role of platelets, being not only applicable to hemostasis and thrombogenesis, but also applicable to enhanced liver function via mediation of liver regeneration. For example, platelet-derived serotonin was observed to contribute not only to cell cycle progression, but also to metabolic pathways important for prevention of hepatic insufficiency [30]. Therefore, it can be seen that lower platelet count has the potential to promote hepatic insufficiency.

A number of limitations were identified with the present study. Firstly, the parameters identified in the fibrosis-4 model may be unstable and influenced by other factors. For example, some drug therapies (such as antineoplastic drugs) or presentation of steatohepatitis prior to hepatectomy has the potential for liver function damage and increased AST/ALT levels. It was observed that whilst some patients reported no previous anti-HBV therapy when diagnosed with HBV-related HCC, other patients described long-term use of anti-HBV therapy, which can influence liver inflammation and liver function. Secondly, the results of the present study are based upon a retrospective analysis. Consequently, it is important to point out that some of the patients in the present study were selected for reasons of surgical safety. Future research should involve additional studies involving larger numbers of patients for a more accurate evaluation of the role of fibrosis-4.

## 5. Conclusion

In conclusion, in accordance with the routine tests that have been performed in daily clinical practice, the marker of fibrosis-4 can accurately predict cirrhosis. Moreover, fibrosis-4 was independent prognostic factor for postoperative hepatic insufficiency, overall survival, and disease-free survival for HCC patients with R0 liver resection.

## Figures and Tables

**Table 1:** Characteristics of the included patients.

Parameters	N(%)
Male	668 (84.8%)
Age (years) (Mean ± SD)	51.1±11.9
Etiology	
HBV	665 (84.4%)
HCV	14 (1.8%)
NBNC	109 (13.8%)
Child-Pugh Score	
A	746 (94.7%)
B	42 (5.3%)

MELD, median (IQR)	8 (7-10)
AST (U/L) (Mean ± SD)	51.1±38.6
ALT (U/L) (Mean ± SD)	49.8±40.6
Total bilirubin (umol/L) (Mean ± SD)	16.2±13.7
Albumin (g/L) (Mean ± SD)	41.2±4.4
INR (Mean ± SD)	1.27±0.15
PLT (109/L) (Mean ± SD)	140±70
Ishak	
0-2	282 (35.8%)
3-4	95 (12.1%)
5-6	411 (52.2%)
Cirrhosis	411 (52.2%)
AFP (ng/mL) (Mean ± SD)	10 (7.4%)
BCLC	
0-A	537 (68.2%)
B	117 (14.8%)
C	134 (17.0%)
Tumor	
Single	636 (80.7%)
Multiple	152 (19.3%)
Macrovascular invasion	93 (11.8%)
Microvascular Invasive	162 (20.6%)
Differentiation Degree	
Poor	89 (11.3%)
Moderate	680 (86.3%)
Well	19 (2.4%)
Recurrent Hepatocellular Carcinoma	37 (4.7%)
Complications III-V	68 (8.6%)
Transfusion	175 (22.2%)
Hospital Stay (days) median (IQR)	9 (7-11)
AST/ALT (Mean ± SD)	1.2±1.0
APRI (Mean ± SD)	1.2±1.7
FIB-4 (Mean ± SD)	3.6±3.7
Lok (Mean ± SD)	12.6±1.6
King Score (Mean ± SD)	33.1±43.7

ALT: alanine aminotransferase; AST: aspartate aminotransferase. AFP: *α*-fetoprotein; BCLC: Barcelona Clinic Liver Cancer; INR: international normalized ratio; PLT: platelet count. APRI: AST-to-platelet ratio Index; HBV: hepatitis B virus; HCV: hepatitis C virus; NBNC: non-B non-C hepatitis virus. MELD: model for end-stage liver disease; IQR: interquartile range; SD: standard deviation.

**Table 2:** Univariate and multivariate analysis of factors associated with overall survival of HCC patients with R0 resection.

Factor	Univariate analysis		Multivariate analysis	
	OR (95 % CI)	P Value	OR (95 % CI)	P Value
Male	0.88 (0.61-1.2)	0.51		
Age	1.0 (0.99-1.01)	0.94		
Etiology				
HBV	0.89 (0.63-1.3)	0.50		
HCV	0.56 (0.18-1.7)	0.31		
Child-Pugh Score				
A	1 (Reference)			
B	1.44 (0.84-2.5)	0.18		
MELD	1.106 (0.93-1.106)	0.721		
Cirrhosis	1.3 (1.01-1.71)	0.04		
AFP	1.0 (1.0-1.0)	0.06		
BCLC				
0-A	1 (Reference)		1(Reference)	
B	1.6 (1.1-1.27)	0.02	1.5(1.04-2.18)	0.03
C	3.65 (2.7-4.9)	<0.01	3.4(2.5-4.66)	<0.01
Tumor				
Single	1 (Reference)			
Multiple	1.52 (1.12-2.1)	<0.01		
Macrovascular invasion	3.55 (2.6-4.8)	<0.01		
Microvascular Invasive	1.9 (1.4-2.5)	<0.01	1.5(1.11-2.01)	<0.01
Differentiation				
Poor	1 (Reference)			
Moderate	0.225 (0.05-1.01)	0.12		
Well	0.813 (0.53-1.24)	0.34		
Recurrent HCC	0.61 (0.30-1.2)	0.17		
Complications III-V	1.7 (1.14-2.54)	0.01	1.6(1.08-2.44)	0.02
Transfusion	1.3 (0.9-1.7)	0.09		
AST/ALT	1.15 (1.07-1.23)	<0.01		
APRI	1.08 (1.02-1.13)	<0.01		
Fibrosis-4	1.04 (1.02-1.07)	<0.01	1.05(1.03-1.08)	<0.01
Lok	1.11 (1.05-1.17)	<0.01		
King Score	1.01 (1.01-1.01)	<0.01		

ALT: alanine aminotransferase; AST: aspartate aminotransferase. AFP: *α*-fetoprotein; BCLC: Barcelona Clinic Liver Cancer; APRI: AST-to-platelet ratio index; HBV: hepatitis B virus; HCV: hepatitis C virus. OR: odds ratio; CI: confidence interval; HCC: hepatoma carcinoma.

**Table 3:** Univariate and multivariate analysis of factors associated with disease free survival of HCC patients with R0 resection.

Factor	Univariate analysis		Multivariate analysis	
	OR (95 % CI)	P Value	OR (95 % CI)	P Value
Male	0.85 (0.63-1.15)	0.293		
Age	0.99 (0.99-1.01)	0.862		
Etiology				
HBV	0.99 (0.74-1.33)	0.965		
HCV	0.54 (0.22-1.31)	0.173		
Child Score				
A	1 (Reference)			
B	1.49 (0.97-2.28)	0.06		

MELD	0.999 (0.914-1.90)	0.989		
Cirrhosis	1.27 (1.03-1.57)	0.02		
AFP	1.00 (1.00-1.00)	0.03		
BCLC				
0-A	1 (Reference)		1(Reference)	
B	1.27 (0.97-1.71)	0.11	1.27(0.95-1.71)	0.11
C	2.73 (2.13-3.50)	<0.01	2.76(2.15-3.56)	<0.01
Tumor				
Single	1 (Reference)			
Multiple	1.31 (1.02-1.68)	0.03		
Macrovascular invasion	2.71 (2.05-3.56)	<0.01		
Microvascular Invasive	1.46 (1.15-1.86)	<0.01		
Differentiation				
Poor	1 (Reference)		1(Reference)	
Moderate	0.67 (0.49-0.93)	0.02	0.69(0.49-0.96)	0.025
Well	0.36 (0.15-0.86)	0.021	0.45(0.19-1.06)	0.066
Recurrent HCC	0.86 (0.53-1.38)	0.521		
Complications III-V	1.38 (0.97-1.94)	0.07		
Transfusion	1.17 (0.91-1.49)	0.224		
AST/ALT	1.04 (1.02-1.06)	<0.01		
APRI	1.07 (1.03-1.12)	<0.01		
Fibrosis-4	1.14 (1.07-1.21)	<0.01	1.05(1.02-1.07)	<0.01
Lok	1.09 (1.04-1.15)	<0.01		
King Score	1.01 (1.001-1.004)	<0.01		

ALT: alanine aminotransferase; AST: aspartate aminotransferase. AFP: *α*-fetoprotein; BCLC: Barcelona Clinic Liver Cancer; INR: international normalized ratio; PLT: platelet count. APRI: AST-to-platelet ratio index; HBV: hepatitis B virus; HCV: hepatitis C virus; NBNC: non-B non-C hepatitis virus. IQR: interquartile range; SD: standard deviation.

**Table 4:** Characteristics of the included patients with fibrosis-4<3.15 and fibrosis-4*⩾*3.15.

Parameters	Fibrosis-4 <3.15	Fibrosis-4 *⩾*3.15	P value
	(N=477)	(N=311)	
Male (%)	411 (86.2%)	257 (82.6%)	0.188
Age (years) (Mean ± SD)	47.7±11.7	56.3±10.4	<0.01
Etiology			
HBV (%)	397 (83.2%)	268 (86.2%)	0.272
HCV (%)	5 (1.0%)	9 (2.9%)	0.094
NBNC (%)	81 (17.0%)	44 (14.1%)	0.319
Child Grade			0.037
A (%)	458 (96.0%)	288 (92.6%)	
B (%)	19 (4.0%)	23 (7.4%)	

MELD, median (IQR)	8 (7-10)	9 (7-10)	0.001
AST (U/L) (Mean ± SD)	39.9±18.5	68.4±52.7	<0.01
ALT (U/L) (Mean ± SD)	44.5±26.8	58.1±54.6	<0.01
Total bilirubin (umol/L) (Mean ± SD)	15.5±16.2	17.3±8.5	0.06
Albumin (g/L) (Mean ± SD)	41.5±4.2	40.7±4.7	0.035
INR (Mean ± SD)	1.26±0.14	1.31±0.15	<0.01
PLT (10^9^/L) (Mean ± SD)	170.2±67.7	94.2±43.6	<0.01
Ishak			<0.01
0-2 (%)	265 (55.6%)	17 (5.5%)	
3-4 (%)	83 (17.4%)	12 (3.9%)	
5-6 (%)	129 (27.0%)	282 (90.7%)	
Cirrhosis (%)	129 (27.0%)	282 (90.7%)	<0.01
AFP (ng/mL) median (IQR)	315 (9.5-1210)	140 (8.6-1210)	0.24
BCLC			0.082
0-A (%)	328 (68.8%)	209 (67.2%)	
B (%)	61 (12.8%)	56 (18%)	
C (%)	88 (18.4%)	46 (14.8%)	
Tumor			0.355
Single (%)	390 (81.8%)	246 (79.1%)	
Multiple (%)	87 (18.2%)	65 (20.9%)	
Macrovascular invasion (%)	59 (12.4%)	34 (10.9%)	0.541
Microvascular Invasive (%)	98 (20.5%)	64 (20.6%)	0.991
Differentiation Degree			0.674
Poor (%)	56 (11.7%)	33 (10.6%)	
Moderate (%)	408 (85.5%)	272 (87.5%)	
Well (%)	13 (2.7%)	6 (1.9%)	
Recurrent Hepatocellular Carcinoma (%)	19 (4.0%)	18 (5.8%)	0.242
Complications III-V (%)	36 (7.5%)	32 (10.3)	0.180
Hepatic Insufficiency (%)	30 (6.3%)	37 (11.9%)	0.006
Transfusion (%)	101 (21.2%)	74 (23.8%)	0.387
Hospital Stay (days) median (IQR)	8 (7-10)	9 (7-11)	<0.01

ALT: alanine aminotransferase; AST: aspartate aminotransferase. AFP: *α*-fetoprotein; BCLC: Barcelona Clinic Liver Cancer; INR: international normalized ratio; PLT: platelet count. APRI: AST-to-platelet ratio index; HBV: hepatitis B virus; HCV: hepatitis C virus; NBNC: non-B non-C hepatitis virus. IQR: interquartile range; SD: standard deviation.

## Data Availability

The data analyzed during this study have been provided in the manuscript, and any further information can be made available from the corresponding author on request.
